# Improvement of antioxidant capacity, aroma quality, and antifungal ability of cherry by phenyllactic acid treatment during low temperature storage

**DOI:** 10.3389/fpls.2024.1529127

**Published:** 2024-12-20

**Authors:** Chaoqi Zhang, Yunfan Wang, Mengxin Wang, Yanhui Kong, Xiulian Li, Danliangmin Song, Xiangquan Zeng, Yanqing Yang, Xinguang Fan, Hansheng Gong

**Affiliations:** ^1^ School of Food Engineering, Yantai Key Laboratory of Nanoscience and Technology for Prepared Food, Yantai Engineering Research Center of Green Food Processing and Quality Control, Ludong University, Yantai, Shandong, China; ^2^ Department of Landscape Construction and Maintenance, Yantai Landscape Construction and Maintenance Center, Yantai, Shandong, China; ^3^ College of Pharmacy, Binzhou Medical University, Yantai, Shandong, China; ^4^ Department of Food Science, College of Agriculture, Purdue University, West Lafayette, IN, United States

**Keywords:** PLA, antioxidant capacity, antifungal ability, cherry, aroma

## Abstract

**Introduction:**

Sweet cherries (*Prunus avium* L.) are highly valued for their taste and nutrients but are prone to decay due to their delicate skin and high respiration rate. Traditional chemical preservatives have drawbacks like residues and resistance, prompting the search for natural alternatives. Phenylactic acid (PLA) has shown promise due to its antibacterial and antioxidant properties, making it a potential natural preservative to extend cherry shelf life.

**Methods:**

'Stella' sweet cherries were treated with varying concentrations of PLA (0, 2, 4, 8, 16 mmol·L^-1^) and stored at 4°C. Key quality indicators, including firmness, total acidity, total soluble solids, weight loss, decay index, and antioxidant activity, were assessed over time. Additionally, HPLC, GC-MS, GC-IMS, colony counts, in vivo inhibition analyses were conducted to evaluate phenolic content, aroma compounds and antifungal ability.

**Results and Discussion:**

PLA at 8 mmol·L^-1^ effectively maintained cherry quality by reducing weight loss and decay of cherries, delaying the decline of firmness, while enhancing antioxidant capacity, flavor stability, and antifungal ability. Higher concentrations (16 mmol·L^-1^) provided stronger antimicrobial effects but caused slight surface wrinkling. Thus, 8 mmol·L^-1^ was optimal, balancing preservation and appearance, making it a promising natural preservative for extending cherry shelf life.

## Graphical Abstract

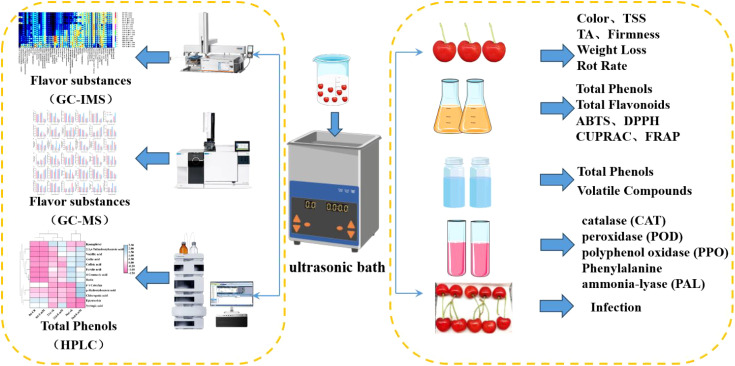

## 1 Introduction

Sweet cherry (*Prunus avium* L.), a prominent member of the *Rosaceae* family and *Prunus* genus, originates from Europe and Western Asia and has now achieved global cultivation (Wani et al., 2024). Sweet cherries are favored by consumers for their delicious taste and rich nutritional components, being high in anthocyanins, organic acids, phenolic compounds, and vitamin C, all of which offer significant health benefits (Wang et al., 2024). However, the thin skin and tender flesh of sweet cherries, along with their high respiration rate, which make them particularly vulnerable to microbial damage during storage and transport, resulting in rot and quality deterioration. Relevant studies have shown that the main causes of postharvest decay of sweet cherries can be attributed to three aspects: metabolic disorder of the fruit tissues, mechanical damage post-harvest, and pathogen invasion (Yang X. et al., 2024). Among these, the impact of pathogenic microorganisms is particularly significant, accounting for over 50% of total losses due to rot (Zhang et al., 2022). Traditional chemical fungicides, such as thiabendazole, while effective in inhibiting microbial growth, pose risks of drug residues, environmental pollution, and microbial resistance with prolonged use (Arabpoor et al., 2021).

Therefore, the search for safe, non-toxic, and natural preservatives has become a primary focus of current research. Additionally, the microorganisms responsible for sweet cherry rot include molds and bacteria, and a single chemical preservative often fails to inhibit both types simultaneously, leading to increased safety risks when multiple preservatives are combined for better efficacy. Thus, developing a non-toxic, residue-free natural antibacterial agent capable of inhibiting both molds and bacteria is particularly important for sweet cherry preservation. In recent years, PLA has garnered attention for its excellent antibacterial and antioxidant properties. As a natural organic acid, PLA effectively inhibits the growth of various pathogenic microorganisms (Ning et al., 2021). And previous studies have found that PLA inhibited the growth of major decay-causing *Mucor* of cherries by destroying *Mucor* cells and inhibiting mitochondrial energy metabolism (C. Zhang et al., 2024). Studies have shown that PLA exerted its antibacterial effects through multiple mechanisms, including disrupting cell walls, inhibiting DNA synthesis, and inducing cell apoptosis (Fan et al., 2022). Moreover, its antioxidant capacity significantly supported the preservation of fruit quality, maintaining the color, flavor, and nutritional components of cherries. Yang Y. et al. (2024) found that PLA enhanced ‘Youyi’ and ‘Tieton’ cherry quality and extended storage period.

This study aims to systematically assess the effects of different PLA concentrations on the occurrence of diseases, fruit firmness, acidity, soluble solid content, antioxidant capacity, and flavor of sweet cherries during storage. Furthermore, exploring the application of PLA in fruit preservation will lay the foundation for developing safe, non-toxic natural preservatives to prolong the storage period of fresh produce.

## 2 Materials and methods

### 2.1 Fruit material

The experimental material was ‘Stella’ sweet cherry, which was harvested in May 2023 from Yantai City, Shandong Province, China. To ensure the consistency of the experimental material, freshly harvested cherries were selected based on criteria of being free from off-odors, rot, pests, mechanical damage, and uniform in size. Immediately following harvest, the cherries were pre-cooled at 0°C for 24 h, then surface disinfected using a 0.01% sodium hypochlorite solution to eliminate surface contaminants and microorganisms, ensuring that all surfaces of the cherries were uniformly exposed to the disinfectant to effectively remove surface contaminants and microorganisms. After disinfection, the cherries were rinsed twice with clean water to eliminate any residual sodium hypochlorite, ensuring that subsequent treatments were not affected by chemical residues.

The PLA(CAS number: 7326-19-4) used in this study was obtained from Merck Corporation (Germany). The experiment comprised five treatment groups: sterile water (control group), 2, 4, 8, and 16 mmol·L^-1^ PLA, with 1500 cherries allocated to each group. The experiment was replicated three times, with each replicate containing 500 cherries. Treatment involved soaking the cherries in the respective solutions for 5 min, followed by air drying and storage at 4°C. On each sampling date, 15 cherries were taken to measure firmness, acidity, soluble solid content, color, weight loss, and decay rate. Additionally, 20 cherries were cut into slices and stored in -80°C refrigerator (quick freezing in liquid nitrogen environment) for analysis of total phenols, total flavonoids, antioxidant capacity, antioxidant enzyme activity, and volatile compounds.

### 2.2 Measurement of basic indicators during storage of sweet cherry

On each sampling day, 15 cherries from each treatment group were randomly selected for measurement of basic indicators. The firmness of the cherries was measured using a Brookfield CT3 Texture Analyzer (Food Technology Corporation, USA) at two symmetrical points along the equator of the cherries, and the average value was recorded. Parameters were set as follows: 2 mm probe, 2 mm·s^-1^ velocity, 2 mm distance and 0.05 N trigger force, with results expressed in Newtons (N).

Total acidity (TA) and total soluble solids (TSS) content in sweet cherries were measured using an acidimeter (ATAGO Co., Ltd., Tokyo, Japan) for acidity and a saccharimeter (ATAGO Co., Ltd., Tokyo, Japan) for sugar content. The cherries were homogenized, and the supernatant was used for measurements, with results expressed as percentages.

A colorimeter (Konica Minolta, Inc., Tokyo, Japan) was used for color measurements. The smooth surface of the cherries was selected for measurement, recording L*, a*, and b* values, with color difference expressed as *ΔE* (Maftoonazad et al., 2008), ΔE*=[(ΔL*)^2^ + (Δa*)^2^ + (Δb*)^2^]^1/2^.

Weight loss of the cherries was measured using a weighing method. The initial weight of the cherries in each packaging was recorded as m_0_ (g), and the weight was measured every 7 d, recorded as m_1_ (g). Weight loss percentage was calculated using the formula: Weight Loss (%) = (m_0_ – m_1_)/m_0_ × 100, with results expressed as percentages (Pang et al., 2023).

From each treatment group, 30 sweet cherry samples were randomly selected and placed in sterilized disposable fruit preservation boxes, stored in a 4°C cold storage. Observations were made weekly to record the incidence of decay (Zheng et al., 2024). The fruit was categorized based on decay severity on a scale of 0 to 5, with the number of fruits at each level recorded. The decay levels were defined as follows: 0 indicates the sweet cherry is completely healthy with no signs of decay; level 1 signifies minor decay spots covering less than 20% of the total fruit surface; level 2 indicates numerous spots with decay comprising 20% to 40% of the surface; level 3 denotes a majority of the surface being decayed, accounting for 40% to 60%; level 4 represents severe decay covering 60% to 80%; and level 5 indicates complete decay with over 80% of the surface affected. Rot index was obtained using the formula: Decay index (%) = ∑ (decay grade ×fruit count at that grade)/(5×total fruit count), where ∑ (decay grade × fruit count at that grade) represents the sum of the products of the number of rotted fruits and their corresponding rot grades, 5 denotes the highest rot grade, and the total number of fruits represents the total sample size.

### 2.3 Determination of phenolic compounds in cherry by HPLC

Phenolic profiles were separated and identified using HPLC (Liu et al., 2015). Extraction involved ultrasonic processing of 1 g cherry powder with 1 mL ethanol (95%) for 1 h, followed by centrifugation at 11,000 × *g* for 20 min at 4°C. The supernatant was analyzed using an HPLC system (LC-20A, Shimadzu, Japan) equipped with a C18 column and a diode array detector (Liang et al., 2023). The mobile phase utilized was composed of water containing 0.1% trifluoroacetic acid (Phase A) and a methanol-acetonitrile blend (Phase B, 1:1). The elution gradient included: from 0 to 15 min, phase B increased from 12% to 25%; between 15 and 25 min, it rose from 25% to 35%; from 25 to 50 min, it progressed from 35% to 55%; followed by an increase from 55% to 65% between 50 and 60 min, and finally reduced back to 12% by 60-70 min. The column temperature was kept at 30°C, injection volume was set at 20 μL, and flow rate at 1 mL·min^-1^, with 280 nm detection wavelength for phenolic quantification based on standard calibration curves.

### 2.4 Determination of cherry total phenols, total flavonoids and antioxidant capacity

The total phenolic content (TPC), total flavonoid content (TFC), and antioxidant capacity were evaluated using the concentrated phenolic extracts. For TPC, 100 μL of extract was mixed with 200 μL Folin-Ciocalteu reagent and 1.0 mL sodium carbonate solution (6%, w/w). After heating at 75°C for 10 min, the absorbance was taken at 760 nm (Fan et al., 2017).

For TFC analysis, 100 μL of extract was combined with 100 μL deionized water, 75 μL of 5% sodium nitrite, followed by 150 μL of 10% AlCl_3_·6H_2_O, and 500 μL NaOH (1.0 mol·L^-1^). The final absorbance was noted at 510 nm.

To evaluate antioxidant potential, four assays were employed: ABTS, DPPH, CUPRAC, and FRAP. In the ABTS assay, 300 μL extract mixed with 500 μL ABTS solution (7.4 mmol·L^-1^), kept in the dark for 10 min, was read at 734 nm, results given in mg TE·g^-1^ (Rumpf et al., 2023).

The DPPH method involved mixing 30 μL extract with 200 μL DPPH solution (0.3 mmol·L^-1^) in ethanol, incubated for one hour at 30°C, and measured at 517 nm, results also in mg TE·g^-1^ (Rumpf et al., 2023).

For the CUPRAC assay, 0.1 mL of extract was blended with 100 μL CuCl_2_ solution (5 mmol·L^-1^), 100 μL ethanol neocuproine (3.75 mmol·L^-1^), and 100 μL CH_3_COONH_4_ buffer (1.0 mol·L^-1^, pH = 7.0), left for 30 min at 25°C in darkness and analyzed at 450 nm (Akar and Burnaz, 2019), expressed as mg TE·g^-1^.

The FRAP assay used 200 μL extract, combined with 100 μL of TPTZ reagent (10 mmol·L^-1^), 1.0 mL of FeCl_3_·6H_2_O solution (20 mmol·L^-1^), and 10 mL of acetate buffer (0.3 mol·L^-1^, pH = 3.6), incubated at 37°C for 5 min, reading absorbance at 593 nm. Results were reported in mg TE·g^-1^ (Liang et al., 2023).

### 2.5 Determination of antioxidant enzyme activities of cherry

The assessment of antioxidant enzyme activities in cherry samples was conducted following the modified protocol of Wang et al. (2020). To evaluate catalase (CAT) and phenylalanine ammonia-lyase (PAL) activities, 5 g of cherry pulp was homogenized in various buffer solutions at 4°C to prepare crude enzyme extracts as follows: The extraction buffer for CAT contained 0.005 mol·L^-1^ dithiothreitol (DTT) and polyvinylpyrrolidone (PVP, 5%) was used as an extraction buffer for CAT enzyme. The extraction buffer for POD included 1 mmol·L^-1^ polyethylene glycol (PEG), polyvinylpolypyrrolidone (PVPP, 4%), and Triton X-100 (1%). The extraction buffer for PAL consisted of PVP (40 g·L^-1^), ethylenediaminetetraacetic acid (EDTA, 0.002 mol·L^-1^), and β-mercaptoethanol (0.005 mol·L^-1^). The tissue homogenates were subjected to centrifugation at 4°C and 12,000 × *g* for 20 min. The supernatants obtained served as crude enzyme extracts for subsequent enzyme activity assays.

To quantify CAT activity, 100 µL of the enzyme extract was added to 2.9 mL of a hydrogen peroxide (H_2_O_2_, 0.02 mol·L^-1^) solution. Absorbance was recorded at 240 nm after a 15-second interval, with CAT activity reported as units per gram of tissue (U·g^-1^).

For POD activity, 200 μL of H_2_O_2_ (0.5 mol·L^-1^) solution was combined with a reaction mixture containing 3 mL of guaiacol (0.025 mol·L^-1^) and 500 μL of the enzyme extract. The absorbance at 470 nm was measured 15 s after the addition, and POD activity was also noted in U·g^-1^.

PAL activity was determined using a reaction system containing 3 mL of borate buffer (50 mmol·L^-1^) and 500 μL of L-phenylalanine solution (0.02 mol·L^-1^) with 500 μL of enzyme extract. A control was prepared using 500 μL of boiled enzyme extract. This setup was incubated at 37°C for one hour, then terminated by adding 100 μL of hydrochloric acid solution (6 mol·L^-1^). Absorbance readings were taken at 290 nm for both the sample and control tubes (Tomoiaga et al., 2023). PAL activity was presented in U·g^-1^.

### 2.6 Analysis of volatile compounds in sweet cherry

#### 2.6.1 GC-MS analysis

Volatile compounds in cherries were extracted and analyzed using solid-phase microextraction (SPME) combined with gas chromatography-mass spectrometry (GC-MS) (Yao et al., 2018). The aroma profile of cherries treated with sterile water and 8 mmol·L^-1^ PLA was examined at 0, 28, and 56 days, with each treatment replicated thrice.

Pitted cherries were snap-frozen using liquid nitrogen, pulverized, and 1 g of the resulting powder was mixed with 1 mL of NaCl saturated solution and 2 µL of a tridecanone at 0.01 mg·mL^-1^. This was transferred into a 15 mL headspace vial, sealed, and stirred at 40°C for 10 min. SPME fibers (PDMS/DVB/CAR 50/30 µm) were exposed to the vial headspace for 30 min, then thermally desorbed at 250°C for 5 min into a Shimadzu Nexis GC-2030 gas chromatograph equipped with a SH Stabilwax column (60 m × 0.25 mm × 0.25 μm) and GCMS-QP2020 NX mass spectrometer. Helium (purity > 99.999%) served as the carrier gas at 2 mL·min^-1^ flow rate. Injection was splitless at 230°C for 8 min. The oven temperature began at 40°C (held for 2 min), ramping to 230°C at 10°C·min^-1^ and held for 10 min. The ion source was set to 230°C with 70 eV ionization energy, and mass scanning was performed in the 34–348 m·z^-1^. Compound identification was based on NIST library matching and retention indices, with quantification conducted using the internal standard method.

#### 2.6.2 HS-GC-IMS analysis

Volatile organic compounds (VOCs) in cherries were executed using headspace-gas chromatography-ion mobility spectrometry (HS-GC-IMS) with a FlavourSpec instrument (Germany) (Gao et al., 2022). The system utilized an MXT-WAX column (15 m × 0.53 mmID × 1.0 µm) and an automatic headspace sampler. 3 mL sample was placed in a headspace vial (20 mL), heated at 65°C for 15 min, and a 500 µL sample was injected in splitless mode using a heated syringe at 70°C. The carrier gas flow in the drift tube started at 2 mL·min^-1^ for 2 min, increasing to 100 mL·min^-1^ and held for 20 min. VOCs were identified through NIST and IMS libraries, and the laboratory analysis viewer (LAV) software in positive mode, based on retention indices (RI) and drift times (DT).

### 2.7 *In vivo* evaluation of antifungal activity against *Mucor*

Cherry fruits were disinfected by soaking in a sodium hypochlorite solution (0.01%) for 10 min, followed by two rinses with clean water and air drying (Wang et al., 2018). A 3 mm-deep, 3 mm-diameter hole was made in the middle of each cherry, into which 10 µL of sterile water (control) or various concentrations of PLA (2, 4, 8, 16, 32, 64, 128 mmol·L^-1^) were added, followed by inoculation with 10 µL of *Mucor racemosus* LD3.0026 spore suspension (1 × 10^6^ spores·mL^-1^). The treated fruits were stored at 4°C, and mold growth was monitored every 7 days. The antifungal efficacy of PLA at different concentrations was assessed by visually observing mold growth, while noting any surface wrinkling at higher PLA concentrations. Each treatment group comprised 60 cherry fruits with three replicates.

### 2.8 Microbial colony count

A 25 g cherry sample was homogenized in 225 mL of physiological saline to prepare a 1:10 dilution. Subsequently, a series of ten-fold serial dilutions was prepared. For each dilution, 1 mL of each dilution was plated onto a 20 mL plate count agar (PCA) medium and mixed thoroughly, with two plates prepared for each dilution. Additionally, 1 mL of the blank dilution was added to two sterile plates to serve as a control. Each plate was incubated (PCA at 36°C for 48 h; PDA at 28°C for 5 d), and colony-forming units (CFUs) were counted. Bacterial plates with 30-300 CFUs and fungal plates with 10-150 CFUs were considered for counts, averaged from duplicates (Gull et al., 2021).

### 2.9 Statistical analysis

Each treatment was conducted with three biological replicates, and experiments were independently repeated thrice. Data analysis was performed using Origin 2022 software, and variance analysis (ANOVA) was conducted using IBM SPSS Statistics 26 software. Duncan’s multiple range test determined significance (P < 0.05). Results are presented as mean ± standard error (SE). Graphical representations were created using GraphPad.

## 3 Results and discussion

### 3.1 The effect of PLA on storage quality of cherry

**Figure 1:**
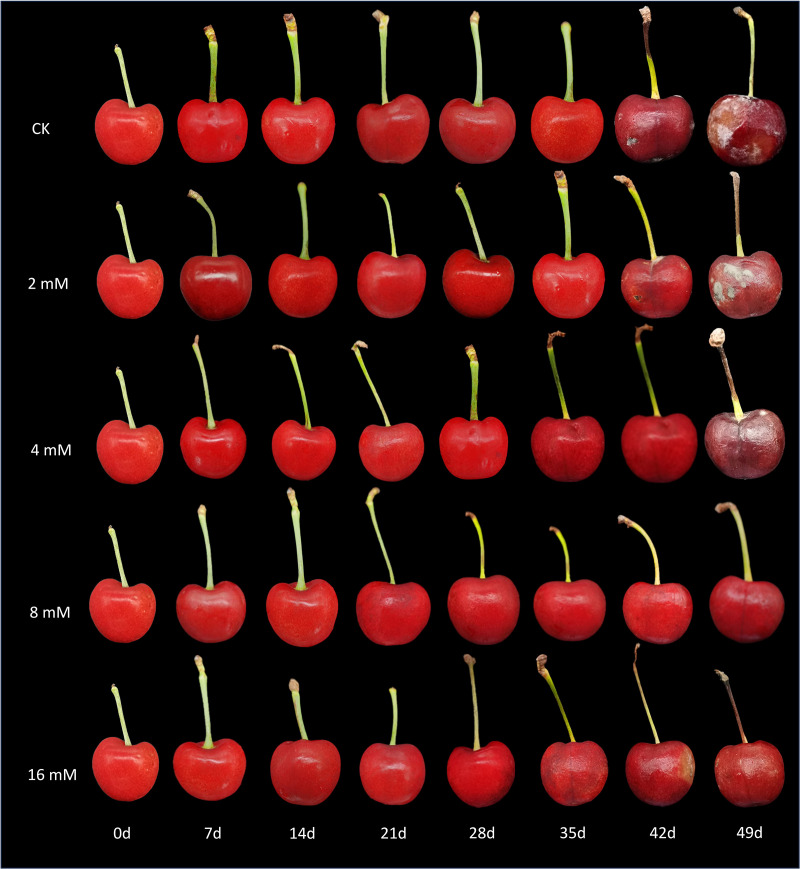
Effect of PLA treatment on the appearance of cherry.

The experimental results indicated that treatment with varying concentrations of PLA significantly reduced the incidence of natural diseases in cherry fruits during storage, demonstrating a concentration-dependent effect (**Figure 1**). At 42 d, both the control group (CK) and the low-concentration PLA treatment groups began to show signs of rot, whereas cherries treated with 8 and 16 mmol·L^-1^ PLA maintained good surface condition with no visible rot. At 49 d, the CK group and the low-concentration treatment groups exhibited worsening rot, while the 8 and 16 mmol·L^-1^ PLA treatment groups showed almost no signs of decay. Although cherries treated with 16 mmol·L^-1^ PLA did not display rot, some surface wrinkling was observed, suggesting that 8 mmol·L^-1^ is the optimal treatment concentration. This phenomenon may be related to the concentration of PLA, which effectively inhibits microbial growth and delays the decay process. Higher concentrations of PLA may enhance the decay resistance of cherries by altering the cell membrane permeability and inhibiting enzyme activity of cherry decay-causing molds (Liu et al., 2018). Therefore, PLA treatment can effectively suppressed the occurrence of diseases in cherry fruits during storage.

**Figure 2:**
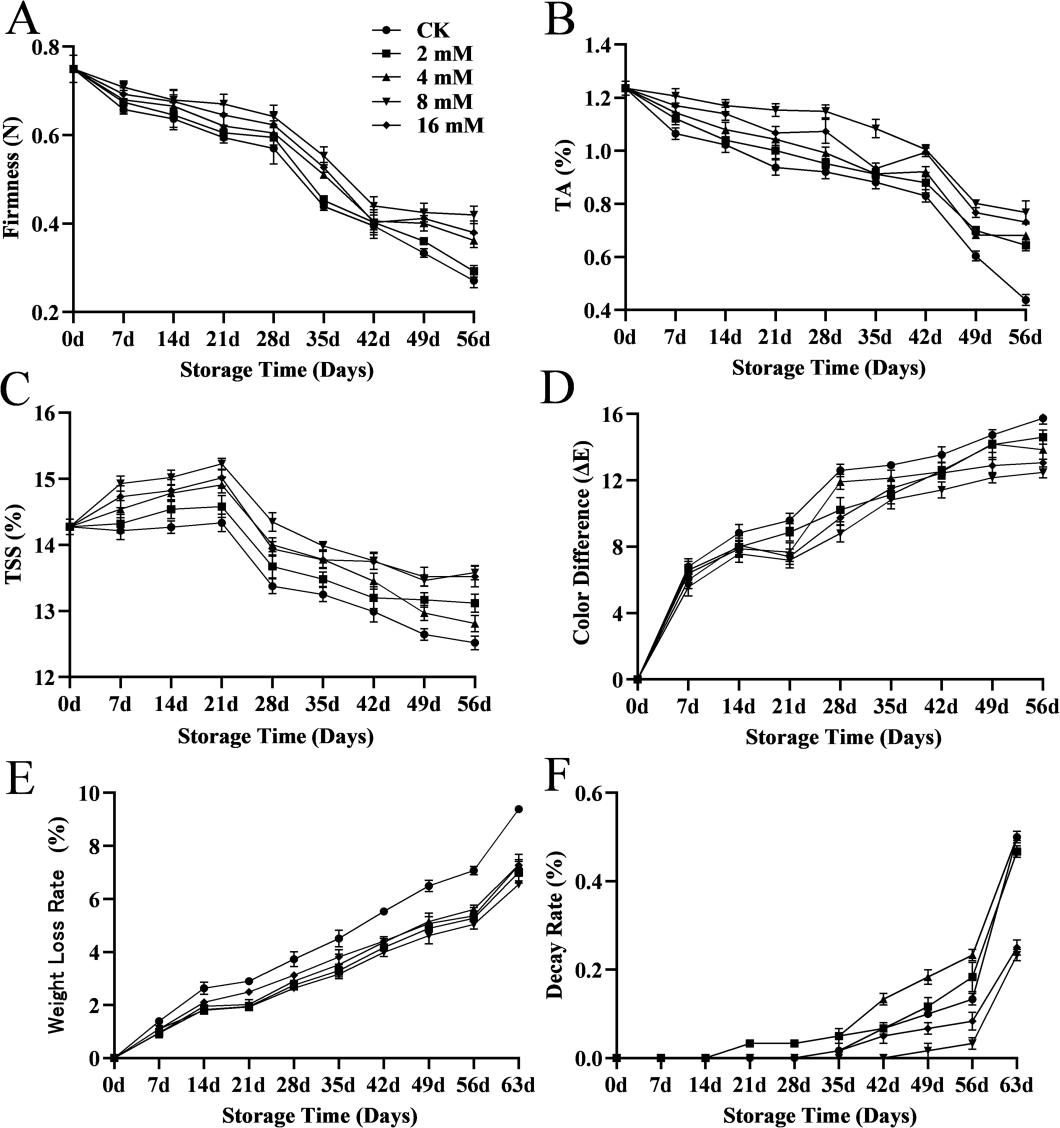
Effect of PLA treatment on cherry firmness **(A)**, titratable acidity **(B)**, soluble solid content **(C)**, color **(D)**, weight loss **(E)**, and decay rate **(F)**.

The firmness of cherries decreased with storage time in all groups, due to fruit ripening and softening (Zeng et al., 2022). The firmness of the 8 mmol·L^-1^ PLA treated group at 56d was 0.41 N, which was much greater than the 0.27N in the CK group (**Figure 2A**). PLA treatments, especially at 8 mmol·L^-1^, significantly slowed the decline in firmness, suggesting a protective effect on the cell wall. Changes in firmness during fruit maturation and storage are associated with cell wall degradation (Bernalte et al., 2003) and various enzymes (such as polygalacturonase, pectin methylesterase, and β-galactosidase) (Zhu et al., 2018). Additionally, prolonged storage resulted in significant moisture loss, causing cherry shrinkage and reduced firmness.

During storage, the acidity of cherries gradually decreased, while PLA treatment was able to mitigate this decline, particularly in the 8 mmol·L^-1^ PLA group, which demonstrated the most significant effect. The acidity of the 8 mmol·L^-1^ PLA treated group at 56 d was 1.65 times that of the CK group (**Figure 2B**). This may be related to the inhibitory effect of PLA on the decomposition and metabolism of organic acids, which allowed the fruit flavor to be maintained during storage. The reduction in titratable acidity (TA) during storage likely reflected metabolic changes in the fruit, as organic acids are consumed during respiration, making the fruit tasted relatively sweeter (Maftoonazad et al., 2008).

Total soluble solids (TSS) is a key indicator of fruit quality, closely related to sugar content and consumer acceptance (Serradilla et al., 2016). The TSS of cherries initially increased during storage before gradually declining, a trend similar to that reported by Zhao et al (Zhao et al., 2019). The slight increase in TSS can be attributed to cell wall degradation, reduced respiration rate in cherries, and increased dry matter due to moisture loss. Changes in soluble solids content may also relate to the conversion of starch to sugar and respiration processes (Zeng et al., 2022). A slight decrease in TSS might occur due to the breakdown of carbohydrates and glycosides during respiration (Khaliq et al., 2019). The soluble solids of cherries in the 8 mmol·L^-1^ PLA treated group at 56 d was 13.58 compared to 12.52 in the CK group (**Figure 2C**). PLA treatment effectively maintained the sugar content of cherries, likely by delaying respiration and sugar metabolism, thereby reducing sugar consumption.

Fruit skin color is a major quality parameter related to consumer acceptance and is often used as a quality indicator (Afonso et al., 2023). Changes in color difference reflected the color changes of cherries during storage, typically showing increased color difference and darker fruit as storage time extends. PLA treatment was found to delay the discoloration and oxidation of cherries, preserving their overall appearance, likely by preventing pigment degradation (**Figure 2D**).

Weight loss is a key factor leading to the deterioration of sweet cherries and affects their shelf life (Bernalte et al., 2003). It is also a significant indicator of moisture loss during storage due to transpiration and respiration processes (Bilgin and Basaran Akocak, 2024). The untreated group exhibited a much higher weight loss rate than the treated groups (**Figure 2E**), indicating that the PLA treatment was able to slow down the evaporative loss of water and maintain the water content of the fruits, which may be related to the protective film formed by PLA on the surface of the fruits, effectively preventing the dissipation of water.

The growth of microorganisms, especially molds and bacteria, is the main cause of fruit rot (Krasniewska et al., 2017). The decay rate at 63 d was 23% in the 8 mmol·L^-1^ PLA treated group and 50% in the CK group (**Figure 2F**). The decay rate of the untreated group gradually increased with time, and the high concentration of PLA treatment was effective in inhibiting cherry decay and prolonging the storage period.

In conclusion, high concentrations of PLA treatments (especially 8 mmol·L^-1^ PLA) significantly slowed down the decline in cherry hardness, maintained better color, delayed the decline in sugar and acidity, and reduced weight loss and decay rates. Therefore, PLA treatment was a highly effective method for preserving cherry freshness and extending shelf life, with the most pronounced effect observed at 8 mmol·L^-1^.

### 3.2 The effect of PLA on phenolic compounds content of cherry

**Figure 3:**
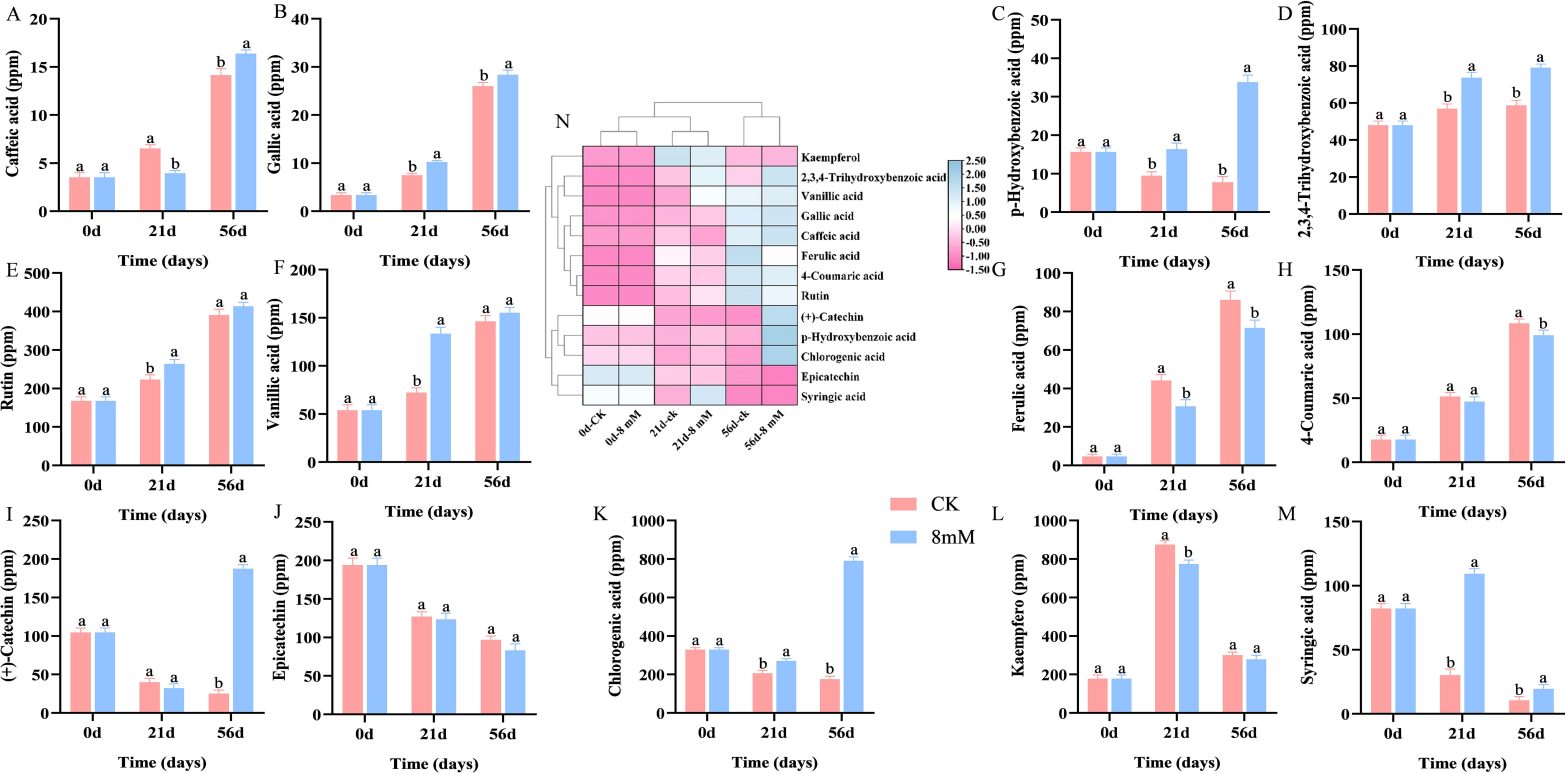
HPLC analysis of the effect of PLA on phenolic compound content in cherry: caffeic acid **(A)**, gallic acid **(B)**, p-hydroxybenzoic acid **(C)**, 2,3,4-trihydroxybenzoic acid **(D)**, rutin **(E)**, vanillic acid **(F)**, ferulic acid **(G)**, 4-coumaric acid **(H)**, (+)-catechin **(I)**, epicatechin **(J)**, chlorogenic acid **(K)**, kaempferol **(L)**, and syringic acid **(M)**, with a clustered heat map of total phenolic content for different PLA concentrations and storage days **(N)**. Statistical significance was determined at p < 0.05 according to a one-way analysis of variance (ANOVA) and Duncan’s test. Different letters represent significant differences at p < 0.05.

Phenolic compounds are key bioactive substances in fruits, contributing to health benefits such as reducing cancer risks and offering neuroprotective properties, and the flavor and astringency of cherries are significantly affected by phenolics (Bernalte et al., 2003). Phenolic compounds contribute to antioxidants and are positively correlated with antioxidant capacity (Mokrani et al., 2016). Antioxidants like phenolics protect against reactive oxygen species (Wang et al., 2015). Treatment with PLA has been shown to increase the accumulation of various phenolic compounds, including caffeic acid (**Figure 3A**), gallic acid (**Figure 3B**), p-hydroxybenzoic acid (**Figure 3C**), 2,3,4-trihydroxybenzoic acid (**Figure 3D**), rutin (**Figure 3E**), vanillic acid (**Figure 3F**), ferulic acid (**Figure 3G**), 4-coumaric acid (**Figure 3H**), (+)-catechin (**Figure 3I**), chlorogenic acid (**Figure 3K**), and kaempferol (**Figure 3L**), while the contents of epicatechin (**Figure 3J**) and syringic acid (**Figure 3M**) decrease. **Figure 3N** shows the clustered heat map of total phenolic content of cherries treated with different PLA concentrations for different storage days. Caffeic acid, 2,3,4-trihydroxybenzoic acid, gallic acid, rutin, vanillic acid, ferulic acid, 4-coumaric acid, and kaempferol clustered together, with their contents gradually increasing as the storage time is extended. (+)-Catechin, chlorogenic acid, p-hydroxybenzoic acid, epicatechin, and syringic acid clustered together, with their contents gradually decreasing as the storage time is extended. With extended storage time, PLA treatment enhanced the retention and stability of various phenolic compounds in cherries, particularly chlorogenic acid, p-hydroxybenzoic acid, and (+)-catechin. Chlorogenic acid, being the most abundant compound, exhibited significant stability under PLA treatment, underscoring its importance as a key phenolic compound that contributes to the antioxidant and flavor characteristics of cherries. PLA helped maintain higher levels of total phenolic content in cherries, which can enhance their nutritional and sensory quality.

### 3.3 The effect of PLA on total phenols, total flavonoids, and antioxidant capacity of cherry

**Figure 4:**
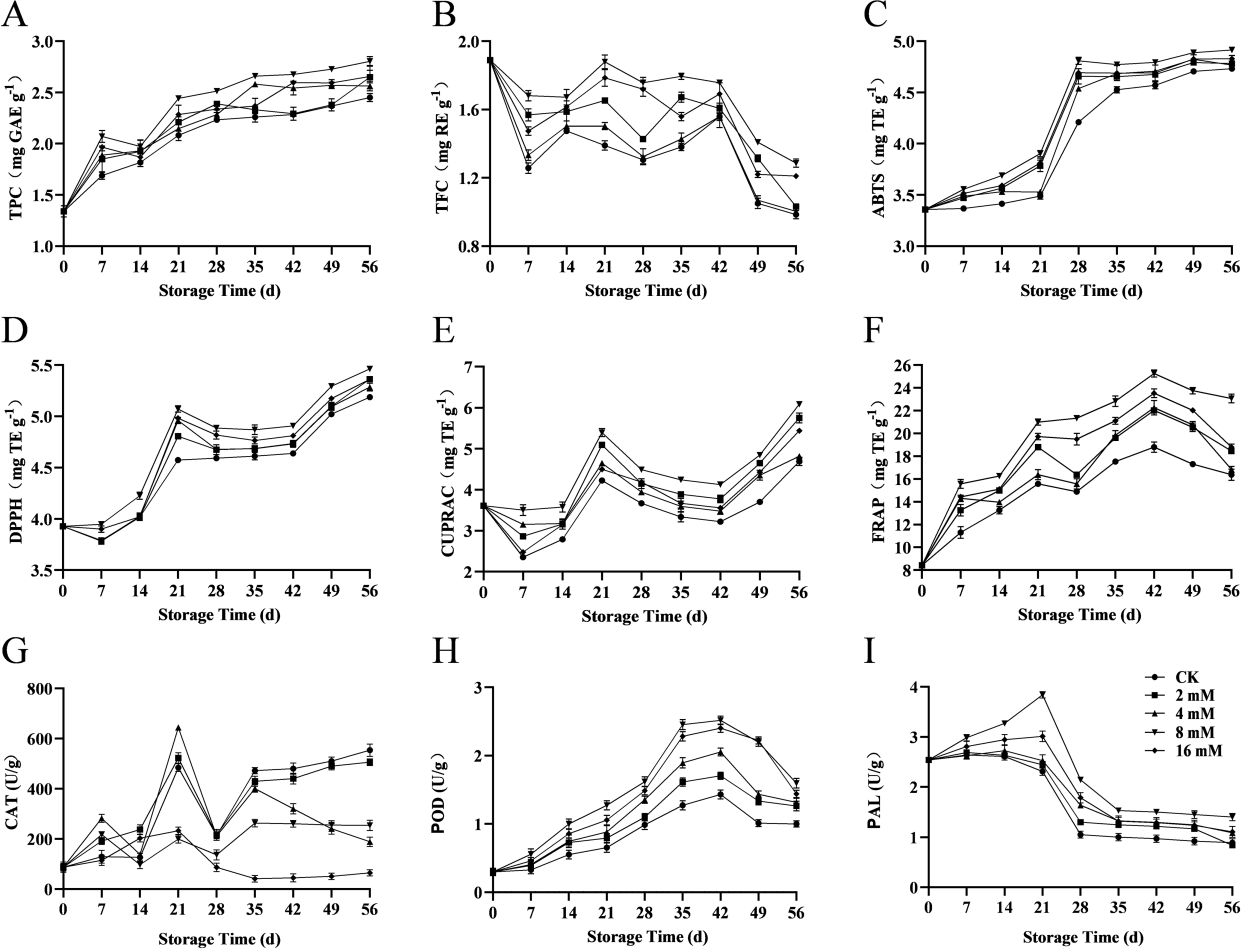
Effect of PLA on total phenolics **(A)**, total flavonoids **(B)**, ABTS **(C)**, DPPH **(D)**, CUPRAC **(E)**, FRAP **(F)**, CAT activity **(G)**, POD activity **(H)**, and PAL activity **(I)** in Cherry.

Sweet cherries are rich in phytochemicals, including phenolics and flavonoids, which enhance fruit quality and nutritional value. Phenolic compounds enhance the color, flavor, aroma, and taste of fruits, thereby improving their quality and nutritional value (Wani et al., 2024). Total phenols, as an important antioxidant, effectively scavenge free radicals and slow down the fruit decay process (Sadiq et al., 2023). In the 8 mmol·L^-1^ PLA treated group, total phenolic content increased from 0.43 mg GAE·g^-1^ at day 0 to 0.78 mg GAE·g^-1^ at day 56 (**Figure 4A**). The data showed that due to the accumulation of secondary metabolites and the ripening process, the total phenolic content generally increased during storage. A similar trend was observed in cherries coated with alginate oil nanoemulsions (Gutierrez-Jara et al., 2021). High concentrations of PLA treatment positively impacted the preservation of phenolic compounds, suggesting its significant role in maintaining the antioxidant capacity of cherries.

Total flavonoids not only contribute to the color of cherries but also possess antioxidant and anti-inflammatory properties. In this study, the 8 mmol·L^-1^ PLA treatment group maintained a high total flavonoid content during the later storage period (**Figure 4B**), indicating that PLA treatment effectively inhibited the degradation of flavonoids, thereby helping to preserve the color, flavor, aroma, and nutritional value of cherries.

ABTS and DPPH assays are commonly used methods to evaluate the antioxidant potential of foods. Experimental results showed that PLA treatment significantly enhanced the ABTS (**Figure 4C**) and DPPH (**Figure 4D**) scavenging ability in cherries, indicating an increase in antioxidant activity. It is hypothesized that it may be closely related to the inhibition of free radical generation and maintenance of antioxidant enzyme activities by PLA, which enhanced the antioxidant capacity of cherries and slowed down the quality decline during storage.

Copper and iron are essential trace elements crucial for the activity of antioxidant enzymes and physiological functions in plants. The experiment demonstrated that the 8 mmol·L^-1^ PLA treatment group had significantly higher copper (**Figure 4E**) and iron (**Figure 4F**) levels during storage than the control group, further confirming the positive role of PLA in preserving mineral content. Maintaining stable levels of these trace elements enhanced the antioxidant defense mechanisms in cherries, thereby improving their preservation effects.

In conclusion, PLA treatment significantly improved the quality of cherries during storage, maintaining high antioxidant capacity and nutritional components. PLA treatment provided an effective strategy to prolong the storage period of cherries and preserved their desirable edible quality by increasing the levels of total phenols and flavonoids while enhancing antioxidant properties.

### 3.4 The effect of PLA on antioxidant enzyme activity of cherry

Catalase (CAT) is an important antioxidant enzyme, and its activity varied significantly across different concentrations of PLA treatment. In this study, CAT activity was influenced by PLA treatment at varying concentrations, with 8 mmol·L^-1^ PLA notably enhancing the antioxidant defense of cherries (**Figure 4G**). However, with prolonged storage time, CAT activity gradually decreased, especially after 28 d, where a notable decline was observed in the 16 mmol·L^-1^ PLA group, suggesting that high concentrations of PLA may inhibit CAT activity.

Among the antioxidant enzymes in plants, peroxidase (POD) is the key redox enzyme involved in the reactive oxygen species (ROS) scavenging system. It plays a role in catalyzing the oxidation of phenolic compounds to quinones, which is responsible for the browning of sweet cherries (Mirto et al., 2018). Peroxidase (POD) plays a crucial role in antioxidant responses. The results indicated an increase in POD activity across all treatment groups, with the 8 and 16 mmol·L^-1^ PLA groups showing the most significant changes (**Figure 4H**). This suggested that high concentrations of PLA may enhance the antioxidant capacity of cherries, effectively delaying the aging process of the fruit. The rise in POD activity helped scavenge reactive oxygen species, reducing oxidative damage and thereby maintaining the freshness and nutritional value of the fruit.

Phenylalanine ammonia hydrolase (PAL) is associated with the synthesis of phenolic compounds and is another important enzyme in the biosynthesis of phenolic compounds. The results indicated that PLA treatment significantly increased PAL activity, particularly in the 8 mmol·L^-1^ PLA treatment group (**Figure 4I**). High concentrations of PLA facilitated PAL activity, thus accelerating the production of phenolic compounds and enhancing the antioxidant capacity of cherries. This finding aligned with the observed increase in total phenolic content, further confirming the positive effect of PLA on the antioxidant properties of cherries.

In summary, PLA treatment significantly enhanced the antioxidant defense system of cherries by modulating the activity of key antioxidant enzymes (such as CAT, POD, and PAL), effectively prolonging their shelf life. Moderate PLA concentrations (8 mmol·L^-1^) exhibited optimal effects in promoting antioxidant enzyme activity and maintaining cherry quality. However, while high concentrations of PLA (16 mmol·L^-1^) showed early beneficial effects, prolonged storage may inhibit the activity of some enzymes and even degraded phenolic compounds. Therefore, the appropriate selection of PLA treatment concentration is crucial for delaying the decline in storage quality and preserving the antioxidant activity of cherries. Future research could further explore the effects of PLA on other antioxidant mechanisms and its application potential in different fruit varieties, providing a more comprehensive theoretical basis for the development of fruit preservation technologies.

### 3.5 The effect of PLA on volatile compounds of cherry

#### 3.5.1 GC-MS analysis

This research explored the variations of volatile organic compounds (VOCs) in cherry samples under different treatments and storage conditions. A total of 36 VOCs were identified by GC-MS, including 10 aldehydes, 8 alcohols, 4 esters, 3 ketones, 1 phenol, 7 acids, and 3 alkanes (**Table 1**). The results indicated that treatment with PLA significantly impacted the synthesis and stability of aroma compounds in cherries, providing a basis for understanding the preservation of aromatic characteristics during storage.

**Table 1:** GC-MS analysis of the effect of PLA on cherry volatile flavor compound.

	Compounds	CAS	Formula	Odor description	Odor Threshold (μg/kg)	Concentration(μg/kg)					
						0 d CK	0 d 8 mM	21 d CK	21 d 8 mM	56 d CK	56 d 8 mM
1	Hexanal	66-25-1	C6H12O	grass, tallow, fat	4.5	36320.00 ± 2.3^c^	36320.00 ± 2.3^c^	43250.00 ± 2.3^b^	43920.00 ± 2.1^a^	15730.00 ± 2.1^d^	12010.00 ± 2.5^e^
2	Octanal	124-13-0	C8H16O	fat, soap, lemon, green	0.7	570.00 ± 3.1^a^	570.00 ± 3.1^a^	490.00 ± 3.2^b^	440.00 ± 3.2^c^	210.00 ± 3.3^d^	160.00 ± 3.2^e^
3	2-Decenal, (E)-	3913-81-3	C10H18O	orange	0.3	330.00 ± 3.2^a^	330.00 ± 3.2^a^	120.00 ± 3.7^d^	180.00 ± 3.2^b^	130.00 ± 3.6^c^	60.00 ± 3.4^e^
4	Nonanal	124-19-6	C9H18O	fat, citrus, green	1	2550.00 ± 3.2^a^	2550.00 ± 3.2^a^	1510.00 ± 3.1^c^	2500.00 ± 3.2^b^	1010.00 ± 3.2^d^	670.00 ± 2.3^e^
5	2,4-Hexadienal, (E,E)-	142-83-6	C6H8O	green	10	1480.00 ± 2.3^c^	1480.00 ± 2.3^c^	1710.00 ± 2.1^b^	2140.00 ± 2.2^a^	600.00 ± 2.2^e^	810.00 ± 2.3^d^
6	Benzaldehyde	100-52-7	C7H6O	almond, burnt sugar	350	10250.00 ± 2.1^e^	10250.00 ± 2.1^e^	10890.00 ± 2.2^d^	14660.00 ± 2.3^c^	211200.00 ± 2.2^a^	69450.00 ± 2.2^b^
7	2,6-Nonadienal, (E,Z)-	557-48-2	C9H14O	cucumber, wax, green	0.01	130.00 ± 2.3^e^	130.00 ± 2.3^e^	140.00 ± 2.3^d^	210.00 ± 2.3^a^	200.00 ± 2.6^b^	150.00 ± 1.7^c^
8	2-Heptenal, (E)-	18829-55-5	C7H12O	soap, fat, almond	13	250.00 ± 1.9^a^	250.00 ± 1.9^a^	180.00 ± 1.8^c^	240.00 ± 1.8^b^	160.00 ± 1.8^d^	140.00 ± 1.4^e^
9	Decanal	112-31-2	C10H20O	soap, orange peel, tallow	0.1	730.00 ± 1.7^b^	730.00 ± 1.7^b^	500.00 ± 1.6^c^	860.00 ± 1.6^a^	310.00 ± 1.8^e^	340.00 ± 1.5^d^
10	2-Hexenal, (E)-	6728-26-3	C6H10O	green, leaf	17	3100.00 ± 1.7^c^	3100.00 ± 1.7^c^	3280.00 ± 2.1^b^	3400.00 ± 2.4^a^	820.00 ± 2.3^e^	1270.00 ± 2.5^d^
11	Propanoic acid, 2-methyl-, 3-hydroxy-2,2,4-trimethylpentyl ester	77-68-9	C12H24O3	–	–	490.00 ± 2.5^a^	490.00 ± 2.5^a^	230.00 ± 2.6^c^	280.00 ± 2.9^b^	150.00 ± 3.1^d^	130.00 ± 3.3^e^
12	2,2,4-Trimethyl-1,3-pentanediol diisobutyrate	6846-50-0	C16H30O4	–	–	2380.00 ± 2.4^c^	2380.00 ± 2.4^c^	1100.00 ± 2.5^d^	4390.00 ± 3.1^a^	580.00 ± 3.2^e^	2470.00 ± 3.2^b^
13	Isopropyl palmitate	142-91-6	C19H38O2	fat	–	80.00 ± 2.8^c^	80.00 ± 2.8^c^	110.00 ± 2.6^b^	190.00 ± 2.5^a^	70.00 ± 2.8^d^	30.00 ± 2.8^e^
14	Dimethyl phthalate	131-11-3	C10H10O4	sl. aromatic odor	–	5430.00 ± 3.2^b^	5430.00 ± 3.2^b^	4590.00 ± 3.1^c^	8990.00 ± 3.2^a^	3460.00 ± 2.9^e^	4320.00 ± 2.7^d^
15	5-Hepten-2-one, 6-methyl-	110-93-0	C8H14O	citrus	50	490.00 ± 2.7^b^	490.00 ± 2.7^b^	390.00 ± 3.2^d^	930.00 ± 3.2^a^	390.00 ± 3.6^d^	460.00 ± 3.1^c^
16	5,9-Undecadien-2-one, 6,10-dimethyl-	689-67-8	C13H22O	–	60	640.00 ± 2.8^d^	640.00 ± 2.8^d^	510.00 ± 2.8^e^	1300.00 ± 2.9^a^	780.00 ± 2.8^c^	850.00 ± 2.6^b^
17	3,5-Octadien-2-one	38284-27-4	C8H12O	fruit, fat, mushroom	–	130.00 ± 2.5^c^	130.00 ± 2.5^c^	80.00 ± 2.7^e^	240.00 ± 2.8^a^	180.00 ± 2.9^b^	110.00 ± 2.6^d^
18	Eugenol	97-53-0	C10H12O2	clove, honey	0.1	90.00 ± 3.1^e^	90.00 ± 3.1^e^	110.00 ± 3.2^d^	140.00 ± 3.4^c^	190.00 ± 3.4^b^	220.00 ± 3.4^a^
19	2-Hexen-1-ol, (E)-	928-95-0	C6H12O	green, leaf, walnut	–	22710.00 ± 3.6^b^	22710.00 ± 3.6^b^	27400.00 ± 3.4^a^	19160.00 ± 3.2^c^	5810.00 ± 3.3^d^	4330.00 ± 3.7^e^
20	1-Octen-3-ol	3391-86-4	C8H16O	mushroom	1	350.00 ± 2.3^c^	350.00 ± 2.3^c^	350.00 ± 2.9^c^	690.00 ± 2.8^a^	580.00 ± 2.6^b^	350.00 ± 2.5^c^
21	1-Hexanol, 2-ethyl-	104-76-7	C8H18O	rose, green	270000	3220.00 ± 3.1^a^	3220.00 ± 3.1^a^	1580.00 ± 3.2^d^	3010.00 ± 3.6^b^	2890.00 ± 3.5^c^	1510.00 ± 3.2^e^
22	1-Heptanol	111-70-6	C7H16O	chemical, green	3	240.00 ± 4.1^a^	240.00 ± 4.1^a^	160.00 ± 4.2^c^	220.00 ± 4.2^b^	140.00 ± 4.6^d^	80.00 ± 4.1^e^
23	1-Octanol	111-87-5	C8H18O	chemical, metal, burnt	110	590.00 ± 2.9^b^	590.00 ± 2.9^b^	450.00 ± 2.8^c^	700.00 ± 2.6^a^	410.00 ± 2.5^d^	220.00 ± 2.4^e^
24	1-Nonanol	143-08-8	C9H20O	fat, green	50	190.00 ± 3.1^b^	190.00 ± 3.1^b^	180.00 ± 3.5^c^	260.00 ± 3.6^a^	160.00 ± 3.2^d^	110.00 ± 3.6^e^
25	Benzyl alcohol	100-51-6	C7H8O	sweet, flower	10000	9970.00 ± 2.5^c^	9970.00 ± 2.5^c^	7900.00 ± 2.8^e^	9430.00 ± 2.3^d^	13770.00 ± 2.5^a^	12790.00 ± 2.5^b^
26	2-Ethyl-1-hexanol	104-76-7	C8H18O	citrus	270000	1460.00 ± 1.8^e^	1460.00 ± 1.8^e^	2120.00 ± 1.8^c^	2010.00 ± 1.5^d^	3280.00 ± 1.8^b^	4410.00 ± 1.5^a^
27	Nonanoic acid	112-05-0	C9H18O2	green, fat	3000	260.00 ± 1.3^a^	260.00 ± 1.3^a^	160.00 ± 1.8^c^	210.00 ± 1.7^b^	120.00 ± 1.7^d^	90.00 ± 1.1^e^
28	2-Hexenoic acid	1191-04-4	C6H10O2	fruity	–	360.00 ± 1.8^a^	360.00 ± 1.8^a^	280.00 ± 1.9^d^	310.00 ± 1.8^b^	210.00 ± 1.8^e^	290.00 ± 1.7^c^
29	Acetic acid	64-19-7	C2H4O2	sour	–	740.00 ± 2.1^a^	740.00 ± 2.1^a^	570.00 ± 2.3^b^	530.00 ± 2.3^c^	370.00 ± 2.5^d^	270.00 ± 2.7^e^
30	Hexanoic acid	142-62-1	C6H12O2	sweat	3000	650.00 ± 3.2^d^	650.00 ± 3.2^d^	530.00 ± 3.1^e^	740.00 ± 2.1^b^	670.00 ± 2.3^c^	750.00 ± 2.2^a^
31	Benzoic acid	65-85-0	C7H6O2	urine	–	560.00 ± 2.2^c^	560.00 ± 2.2^c^	290.00 ± 2.5^e^	360.00 ± 2.8^d^	620.00 ± 2.6^b^	700.00 ± 2.7^a^
32	Dodecanoic acid	143-07-7	C12H24O2	Fatty, coconut oil	10000	170.00 ± 2.3^b^	170.00 ± 2.3^b^	130.00 ± 2.6^d^	250.00 ± 2.5^a^	150.00 ± 2.8^c^	80.00 ± 2.3^e^
33	2-methylpropanoic acid,	6228-78-0	C12H24O3	–	–	410.00 ± 2.2^a^	410.00 ± 2.2^a^	230.00 ± 2.7^c^	280.00 ± 1.7^b^	150.00 ± 1.8^d^	130.00 ± 1.9^e^
34	Tetradecane	629-59-4	C14H30	alkane	–	410.00 ± 2.3^a^	410.00 ± 2.3^a^	260.00 ± 2.5^d^	330.00 ± 2.4^b^	210.00 ± 1.9^e^	290.00 ± 1.2^c^
35	Hexadecane	544-76-3	C16H34	alkane	–	160.00 ± 1.3^d^	160.00 ± 1.3^d^	150.00 ± 1.8^e^	210.00 ± 1.2^c^	430.00 ± 1.9^a^	240.00 ± 1.8^b^
36	Pentadecane	629-62-9	C15H32	alkane	–	520.00 ± 2.1^a^	520.00 ± 2.1^a^	470.00 ± 2.3^b^	520.00 ± 1.9^a^	290.00 ± 2.3^d^	320.00 ± 2.4^c^

Odor description: Data sourced from the website https://www.flavornet.org. All values are presented as the mean ± standard deviation (SD). Means with different letters (a-e) in the same row are significantly different (*P* < 0.05).

**Figure 5:**
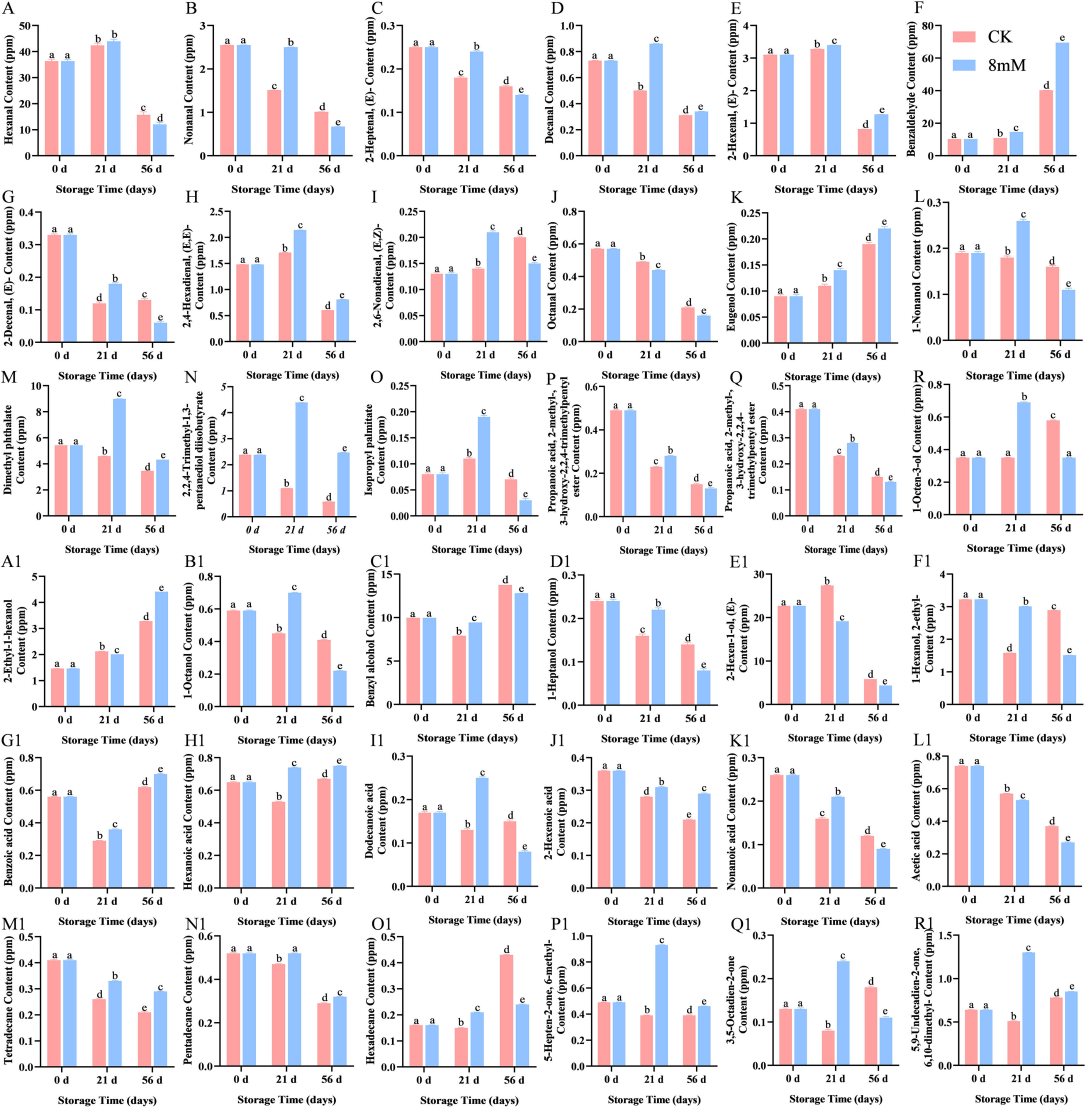
GC-MS analysis of the effect of PLA on cherry volatile compounds: hexanal **(A)**, nonanal **(B)**, (E)-2-heptenal **(C)**, decanal **(D)**, (E)-2-hexenal **(E)**, Benzaldehyde **(F)**, Eugenol **(G)**, hexanoic acid **(H)**, 2,6-nonadienal **(I)**, octanal **(J)**, Eugenol **(K)**, 1-Nonanol **(L)**, dimethyl phthalate **(M)**, 2,2,4-trimethyl-1,3-pentanediol diisobutyrate **(N)**, isopropyl palmitate **(O)**, 2-methylpropanoic acid 3-hydroxy-2,2,4-trimethylpentyl ester **(P)**, Propanoic acid, 2-methyl-, 3-hydroxy-2,2,4-trimethylpentyl ester **(Q)**, 1-Octen-3-ol **(R)**, 2-ethyl-1-hexanol **(A1)**, 1-Octanol **(B1)**, benzyl alcohol **(C1)**, 1-heptanol **(D1)**, **(E)**-2-hexen-1-ol **(E1)**, and 1-hexanol **(F1)**, benzoic acid **(G1)**, Hexanoic acid **(H1)**, lauric acid **(I1)**, 2-hexenoic acid **(J1)**, nonanoic acid **(K1)**, Acetic acid **(L1)**, tetradecane **(M1)**, pentadecane **(N1)**, hexadecane **(O1)**, 6-methyl-5-hepten-2-one **(P1)**, 3,5-octadien-2-one **(Q1)**, 6,10-dimethyl-5,9-undecadien-2-one **(R1)**. Statistical significance was determined at p < 0.05 according to a one-way analysis of variance (ANOVA) and Duncan’s test. Different letters represent significant differences at p < 0.05.

The odor activity value (OAV) for the volatile compounds in cherries were calculated to identify the most characteristic volatiles. The top 10 characteristic volatiles with OAV > 1 (**Table 2**) included 2,6-nonadienal(E,Z)-(13000.00 < OAV < 21000.00), hexanal(2668.89< OAV <9760.00), and decanal(3100.00< OAV <8600.00). Similarly, they are thought to be unique volatiles (Zhang et al., 2024). The alcohols identified included 1-octanol (**Figure 5L**), 1-octen-3-ol (**Figure 5R**), 2-ethyl-1-hexanol (**Figure 5A1**), 1-Octanol (**Figure 5B1**), benzyl alcohol (**Figure 5C1**), 1-heptanol (**Figure 5D1**), (E)-2-hexen-1-ol (**Figure 5E1**), and 1-hexanol (**Figure 5F1**), which contributed to the aroma and flavor of cherries. Among these, benzyl alcohol was found to have the highest concentration, contributing significantly to the aroma and flavor of cherries (Ganatsios et al., 2019). PLA treatment effectively regulated the concentration of alcohols, enhancing the stability of certain alcohols and thereby helping to retain the original flavor of cherries. Specifically, PLA treatment at 8 mmol·L^-1^ improved antioxidant properties, delaying the degradation of flavor components and enhancing the overall quality of cherries. Most ketones contribute pleasant flavors to foods, such as fruits and creams (Li et al., 2021). The results showed that three ketones were generated in the PLA treatment group: 6-methyl-5-hepten-2-one (**Figure 5P1**), 3,5-octadien-2-one (**Figure 5Q1**), and 6,10-dimethyl-5,9-undecadien-2-one (**Figure 5R1**), all of which are typically associated with fruity aromas. This suggested that PLA may positively influence the synthesis and degradation of ketones, further enriching the aromatic profile of cherries. The content of aldehydes is crucial for the flavor and consumer acceptance of foods. Higher concentrations of aldehydes may impart off-flavors, whereas lower concentrations are associated with pleasant flavors. Aldehydes contribute to a range of aromatic, including those associated with almond, citrus, green grass, and floral (Guo et al., 2021).

**Table 2:** Odor active value (OAV) of volatile flavor compounds in cherry.

	Compounds	Odor Threshold (μg/kg)	OAV					
			0 d CK	0 d 8 mM	21 d CK	21 d 8 mM	56 d CK	56 d 8 mM
1	Hexanal	4.5	8071.11	8071.11	9611.11	9760.00	3495.56	2668.89
2	Octanal	0.7	814.29	814.29	700.00	628.57	300.00	228.57
3	2-Decenal, (E)-	0.3	1100.00	1100.00	400.00	600.00	433.33	200.00
4	Nonanal	1	2550.00	2550.00	1510.00	2500.00	1010.00	670.00
5	2,4-Hexadienal, (E,E)-	10	148.00	148.00	171.00	214.00	60.00	81.00
6	Benzaldehyde	350	29.29	29.29	31.11	41.89	603.43	198.43
7	2,6-Nonadienal, (E,Z)-	0.01	13000.00	13000.00	14000.00	21000.00	20000.00	15000.00
8	2-Heptenal, (E)-	13	19.23	19.23	13.85	18.46	12.31	10.77
9	Decanal	0.1	7300.00	7300.00	5000.00	8600.00	3100.00	3400.00
10	2-Hexenal, (E)-	17	182.35	182.35	192.94	200.00	48.24	74.71
11	Propanoic acid, 2-methyl-, 3-hydroxy-2,2,4-trimethylpentyl ester	–	–	–	–	–	–	–
12	2,2,4-Trimethyl-1,3-pentanediol diisobutyrate	–	–	–	–	–	–	–
13	Isopropyl palmitate	–	–	–	–	–	–	–
14	Dimethyl phthalate	–	–	–	–	–	–	–
15	5-Hepten-2-one, 6-methyl-	50	9.80	9.80	7.80	18.60	7.80	9.20
16	5,9-Undecadien-2-one, 6,10-dimethyl-	60	10.67	10.67	8.50	21.67	13.00	14.17
17	3,5-Octadien-2-one	–	–	–	–	–	–	–
18	Eugenol	0.1	900.00	900.00	1100.00	1400.00	1900.00	2200.00
19	2-Hexen-1-ol, (E)-	–	–	–	–	–	–	–
20	1-Octen-3-ol	1	350.00	350.00	350.00	690.00	580.00	350.00
21	1-Hexanol, 2-ethyl-	270000	0.01	0.01	0.01	0.01	0.01	0.01
22	1-Heptanol	3	80.00	80.00	53.33	73.33	46.67	26.67
23	1-Octanol	110	5.36	5.36	4.09	6.36	3.73	2.00
24	1-Nonanol	50	3.80	3.80	3.60	5.20	3.20	2.20
25	Benzyl alcohol	10000	1.00	1.00	0.79	0.94	1.38	1.28
26	2-Ethyl-1-hexanol	270000	0.01	0.01	0.01	0.01	0.01	0.00
27	Nonanoic acid	3000	0.09	0.09	0.05	0.07	0.04	0.03
28	2-Hexenoic acid	–	–	–	–	–	–	–
29	Acetic acid	–	–	–	–	–	–	–
30	Hexanoic acid	3000	0.22	0.22	0.18	0.25	0.22	0.25
31	Benzoic acid	–	–	–	–	–	–	–
32	Dodecanoic acid	10000	0.02	0.02	0.01	0.03	0.02	0.01
33	2-methylpropanoic acid	–	–	–	–	–	–	–
34	Tetradecane	–	–	–	–	–	–	–
35	Hexadecane	–	–	–	–	–	–	–
36	Pentadecane	–	–	–	–	–	–	–

OT: Data primarily sourced from https://www.vcf-online.nl/VcfHome.cfm. OT refers to the odor threshold of volatile flavor compounds, while OAV represents the odor active value of volatile flavor compounds.

PLA treatment increased the levels of hexanal (**Figure 5A**), nonanal (**Figure 5B**), (E)-2-heptenal (**Figure 5C**), decanal (**Figure 5D**), (E)-2-hexenal (**Figure 5E**), and 2,6-nonadienal (**Figure 5I**) in cherry samples. As storage time increased, the levels of these compounds gradually decreased; however, PLA treatment delayed the degradation of aldehydes, maintaining the flavor characteristics of cherries. Aromatic ester compounds are known for their sweet and fruity flavors. The identified esters in cherries included dimethyl phthalate (**Figure 5M**), 2,2,4-trimethyl-1,3-pentanediol diisobutyrate (**Figure 5N**), isopropyl palmitate (**Figure 5O**), and 2-methylpropanoic acid 3-hydroxy-2,2,4-trimethylpentyl ester (**Figure 5P**). These esters play an important role in cherries, providing health benefits, antioxidant activity, and flavor formation (Ghamry et al., 2021). PLA treatment effectively delayed the reduction of ester concentrations, further promoting flavor retention in cherries. PLA treatment could also promote or delay the synthesis or degradation of various acid compounds, including benzoic acid (**Figure 5G1**), hexanoic acid (**Figure 5H**), lauric acid (**Figure 5I1**), 2-hexenoic acid (**Figure 5J1**), and nonanoic acid (**Figure 5K1**). Eugenol (**Figure 5G**), which possesses a spicy aroma, may also have its synthesis promoted by PLA treatment. Additionally, the stability of tetradecane (**Figure 5M1**), pentadecane (**Figure 5N1**), and hexadecane (**Figure 5O1**) was enhanced in the PLA treatment group, which was related to the fatty flavor of the fruit, demonstrating the potential of PLA in maintaining fruit flavor.

#### 3.5.2 HS-GC-IMS analysis

**Figure 6:**
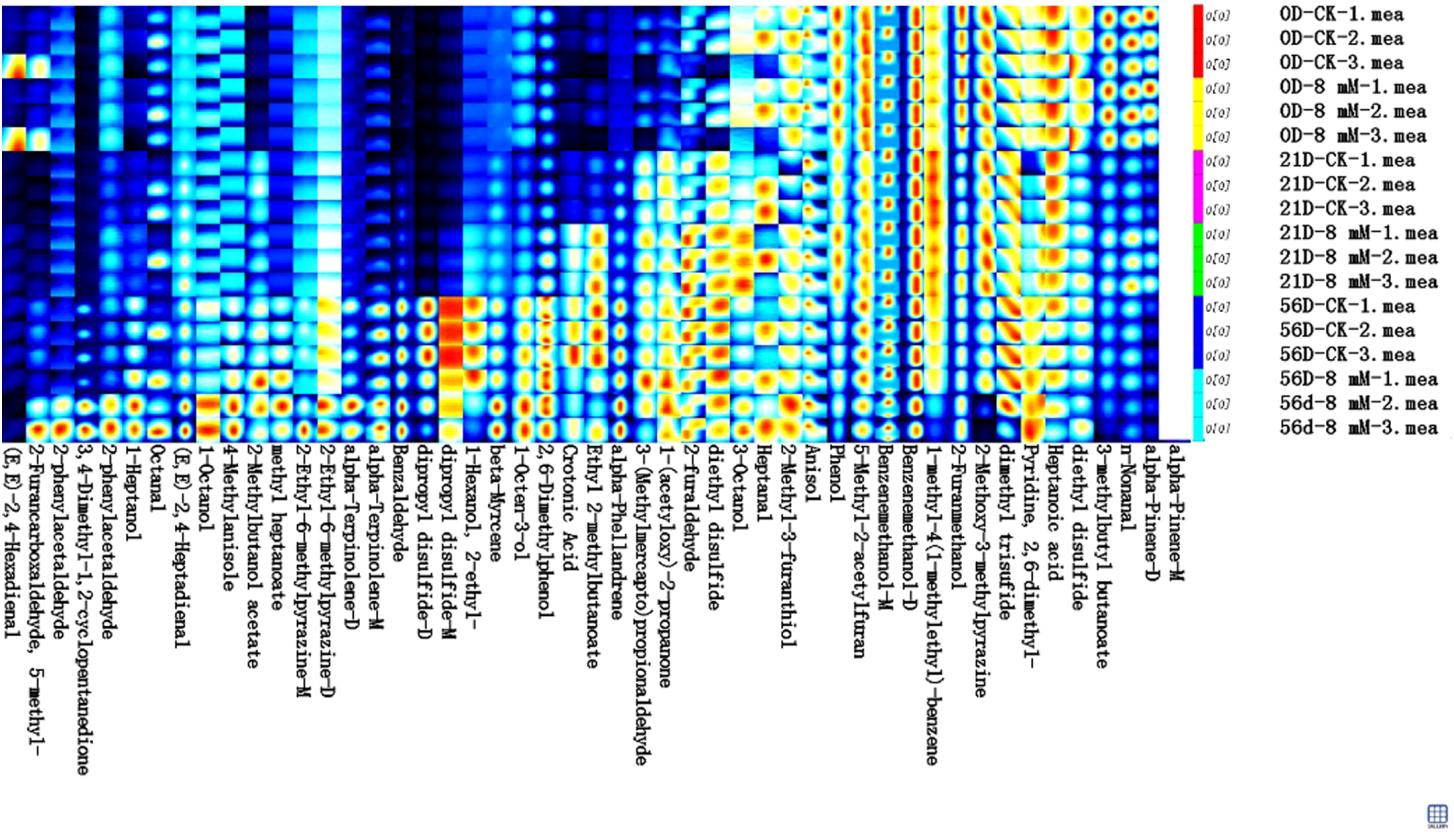
GC-IMS analysis of the effect of PLA on cherry volatile compounds.

In this study, the volatile flavor substances in cherries were systematically analyzed using GC-IMS technology, revealing significant changes in these substances during fruit maturation and storage. The flavor compounds in cherries primarily included aldehydes, alcohols, ketones, esters, sulfides, and heterocyclic compounds (**Figure 6**). Notably, aldehydes such as 2-phenylacetaldehyde and (E,E)-2,4-hexadienal contribute distinct fruity and floral notes, which are essential for the fresh aroma of cherries; alcohols like 1-heptanol and 1-octanol provide mild fruity fragrances; ketones like 3,4-dimethyl-1,2-cyclopentanedione impart creamy or caramelized flavor, enhancing the sweetness of cherries; esters such as methyl heptanoate and 2-methylbutanol acetate add sweet fruity scents, forming an important part of the unique cherry aroma; while sulfur compounds (diethyl disulfide and dimethyl trisulfide) and heterocyclic compounds (2,6-dimethylphenol and alpha-terpinolene) contribute to the complexity and richness of flavor. As the storage period extended to 56 d, especially in the 8 mmol·L^-1^ PLA treatment group, the content of flavor compounds such as (E,E)-2,4-hexadienal and 2-phenylacetaldehyde significantly increased. This increase may be associated with the antibacterial and antioxidant properties of PLA, which effectively inhibited enzymatic reactions and oxidative processes within the fruit, promoting the accumulation of flavor components and resulting in a more intense flavor and fuller aroma of cherries during late storage. Furthermore, PLA treatment slowed the degradation of certain volatile substances, maintaining their higher levels. However, some compounds, such as 3-(methylmercapto) propionaldehyde, 1-(acetyloxy)-2-propanone, 2-furaldehyde, and diethyl disulfide, showed slight reductions after 56 d of storage. The antibacterial effect of PLA inhibited microbial growth, thereby delaying the degradation of these flavor components and maintaining their levels throughout the storage period. Additionally, some components that were initially abundant, such as diethyl disulfide and n-nonanal, began to decrease significantly after 21 d of storage, but PLA treatment effectively extended their retention time.

GC-IMS provided more detailed insights into the changes of low-concentration small molecule aroma compounds in cherries, with higher resolution. This aspect is rarely reported in other studies, further expanding the understanding of cherry aroma. GC-IMS and GC-MS, as complementary analytical techniques, allow for detailed analysis of the volatile flavor compounds in cherries from different perspectives and molecular ranges. GC-IMS excels in detecting low-concentration small molecules and real-time monitoring, while GC-MS is better suited for detecting high-concentration larger molecules and complex aromatic components. By combining these two techniques, a more comprehensive understanding of how PLA treatment regulates the preservation of cherry flavor can be achieved, providing scientific evidence for future fruit preservation and flavor optimization strategies. Similarly, Based on HS-GC-IMS results, the sensitivity of HS-SPME-GC-MS and HS-GC-IMS to these volatile compounds differed due to their distinct identification capabilities (Zeng et al., 2019). This discrepancy may stem from the fact that GC-IMS predominantly detects low-concentration small molecules (C2 ~ C10), while GC-MS identifies high-concentration larger molecules (C7 ~ C13) (Zhang et al., 2024).

In conclusion, both GC-IMS and GC-MS findings indicated that PLA treatment can promote the synthesis of specific aldehydes, alcohols, and esters, delay the loss of specific compounds, thereby preserving the flavor characteristics of cherries. Moreover, the results suggested that, with prolonged storage, certain key flavor compounds exhibited greater stability in the PLA treatment group, supporting the positive role of PLA in maintaining flavor during storage. This provided a new strategy for the storage and flavor retention of cherries, with important implications for research on the preservation of fruits and vegetables.

### 3.6 *In vivo* antifungal effect analysis

**Figure 7:**
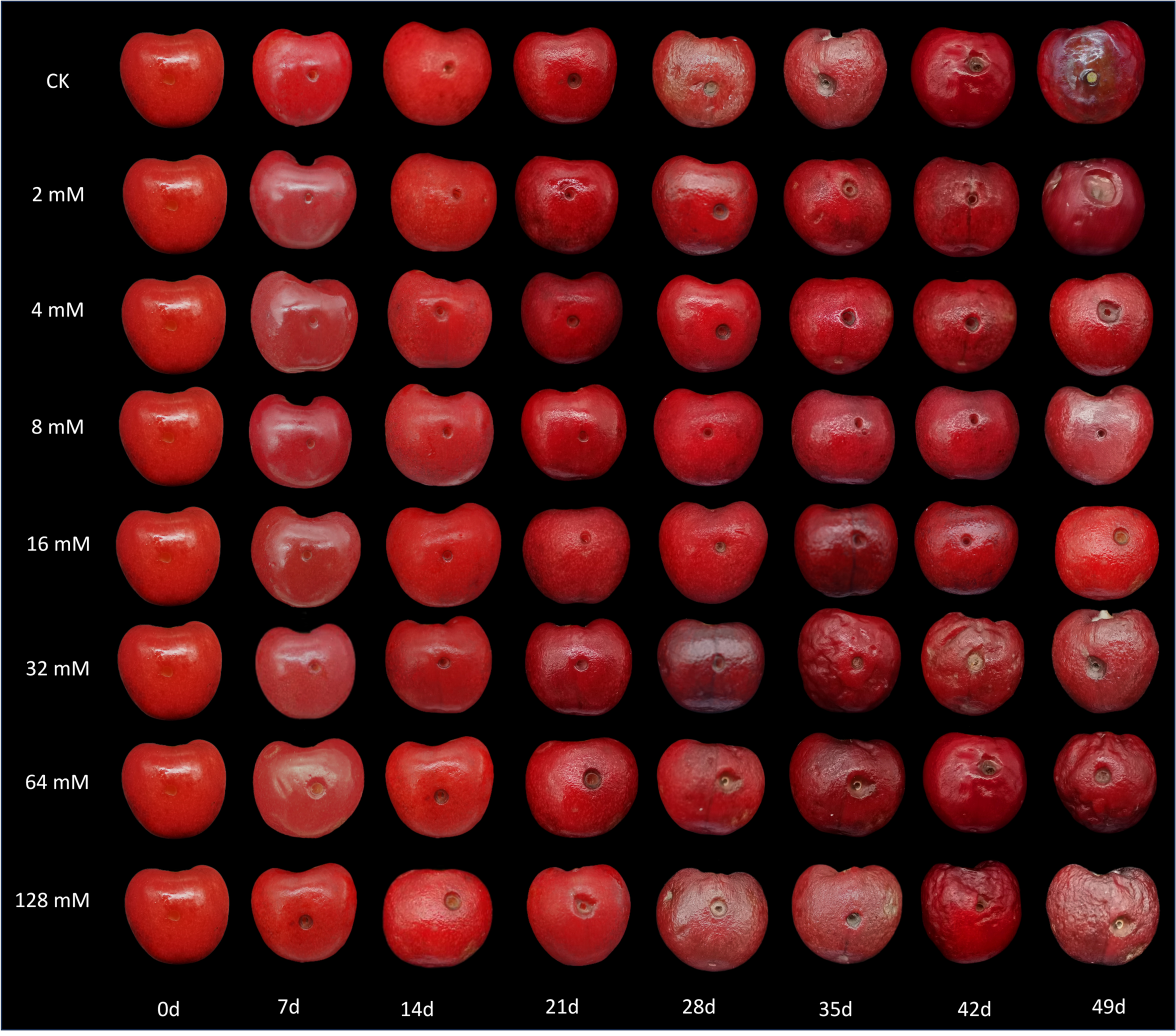
Analysis of the effect of PLA on the *in vivo* resistance of cherry to *Mucor*.

This research explored the influence of varying concentrations of PLA on mold growth on cherries after inoculation. Following puncturing and inoculating cherries with mold, the results showed that PLA significantly inhibited mold growth. Lower concentrations (2 mmol·L^-1^) effectively suppressed early mold growth; however, with prolonged storage time, mold continued to expand gradually. At higher PLA concentrations (8 mmol·L^-1^), the expansion rate of mold on the cherry surface significantly slowed, and at higher concentrations, visible mold growth was almost undetectable (**Figure 7**). This indicated that the inhibitory effect of PLA on mold growth was concentration-dependent.

The inhibitory mechanism of PLA may involve its disruption of the mold cell wall and inhibition of its metabolic activity. High concentrations of PLA can effectively penetrate the mold cell membrane, suppressing its cellular functions, thereby significantly reducing mold reproduction and growth. However, while high concentrations of PLA effectively inhibited mold growth, they also led to surface wrinkling of cherries, affecting the aesthetic quality of the fruit. This wrinkling may result from the high permeability of PLA causing water loss from the cherry skin cells, altering osmotic pressure. Therefore, an optimal PLA concentration (8 mmol·L^-1^) should be selected for cherry preservation to achieve the best balance between inhibiting mold growth and maintaining the appearance of cherries.

### 3.7 Total colony count

**Figure 8:**
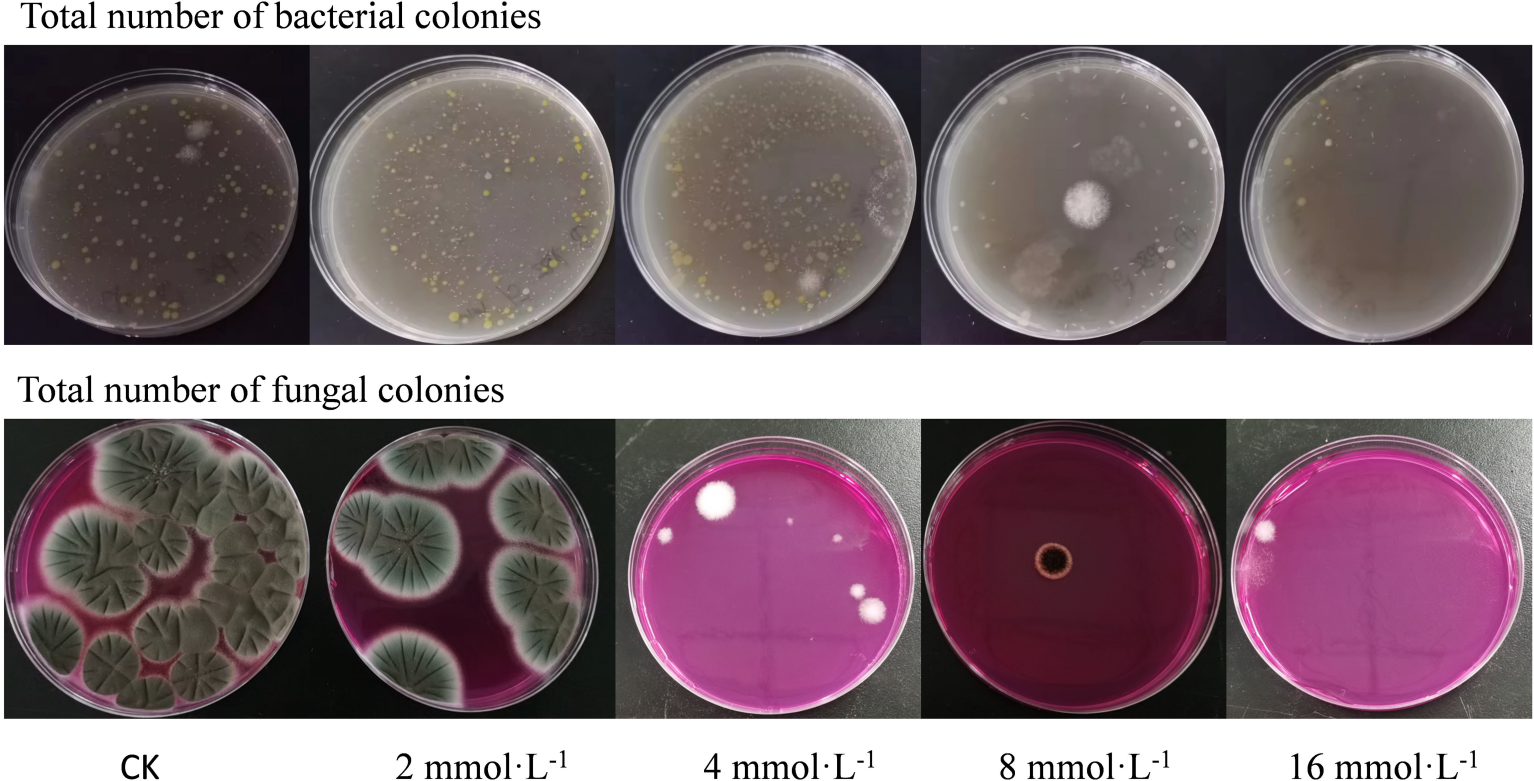
Effect of PLA treatment on total colony count in cherry.

The total bacterial and fungal colony counts on the surface of cherries under different treatment conditions were measured. The results indicated that the untreated control group (CK group) had the highest colony count, while the 16 mmol·L^-1^ PLA treatment group exhibited the lowest count (**Figure 8**). This demonstrated that PLA significantly inhibited the growth of bacteria and fungi on the surface of cherries.

Cherries naturally contain a high moisture content and nutrients, providing a favorable environment for microbial growth. Environmental conditions such as temperature and humidity during storage further promoted microbial proliferation. Without preservation measures, microorganisms on the surface of cherries rapidly grew and reproduced, leading to a decline in fruit quality and a propensity for rot. In contrast, the PLA treated groups displayed a significant antibacterial effect, especially in the 16 mmol·L^-1^ treatment group, which had the lowest colony count, indicating that high concentrations of PLA effectively inhibit the growth of bacteria and fungi.

The antifungal mechanism of PLA may involve its disruption of microbial cell walls and membranes, which reduces microbial growth and reproductive potential (Fang et al., 2022). Thus, the selection of an appropriate PLA concentration is crucial for delaying cherry spoilage, maintaining appearance quality, and extending shelf life.

As highlighted in our results, PLA not only directly inhibited microbial growth but also significantly improved the antioxidant defense system of cherries. The data presented showed that PLA treatment, particularly at concentrations of 8 mmol·L^-1^, significantly increased the activity of key antioxidant enzymes such as CAT, POD, and PAL. Higher PLA concentration (8 mmol·L^-1^) could increase PAL activity, accelerate the synthesis of phenolic compounds, improve the overall antioxidant capacity of fruits, and maintain the quality of fruits during storage. The dual antibacterial and antioxidant effects of PLA make it an effective fresh-keeping agent for cherries after harvest. In our study, 8 mmol·L^-1^ PLA significantly reduced the total colony count of bacteria and fungi on the cherry surface, and also helped to improve antioxidant activity, resulting in better fruit preservation. In conclusion, we believe that PLA’s combination of antimicrobial and antioxidant properties plays a significant role in enhancing cherry preservation by reducing microbial spoilage and maintaining the fruit’s quality during storage. We are confident that this integrated approach to fruit preservation will provide a valuable foundation for the development of more effective post-harvest treatments for cherries and other fruits.

## 4 Conclusion

The concentration of PLA at 8 mmol·L^-1^ had a significant effect on storage quality, antioxidant capacity and flavor stability of cherries. This concentration effectively slowed down the changes in firmness, total acidity, and soluble solids content, while also reducing weight loss and decay, highlighting its role in enhancing post-harvest quality. Furthermore, PLA treatment boosted antioxidant activity, as evidenced by assays such as ABTS, DPPH, CUPRAC, and FRAP, and promoted the activity of key antioxidant enzymes, thus mitigating oxidative damage and prolonging the freshness of cherries. GC-MS and GC-IMS analyses indicated that PLA treatment helped preserve important volatile compounds associated with cherry aroma (benzyl alcohol, hexanal, 2,6-nonadienal (E,Z)-, nonanal), promoting the accumulation of certain flavor compounds while slowing the loss of others, thus maintaining desirable flavor characteristics. Although higher concentrations (16 mmol·L^-1^) effectively inhibited microbial growth, they may lead to slight wrinkling of the cherry surface. Therefore, 8 mmol·L^-1^ represented an optimal balance between antimicrobial efficacy and fruit appearance. In conclusion, PLA at 8 mmol·L^-1^ demonstrated excellent antifungal and antioxidant properties, serving as a promising natural preservative for extending the storage life and enhancing the quality of cherries, providing an effective method for cherry preservation.

## Data Availability

The datasets presented in this study can be found in online repositories. The names of the repository/repositories and accession number(s) can be found in the article/supplementary material.
